# Donepezil Brain and Blood Pharmacokinetic Modeling after Nasal Film and Oral Solution Administration in Mice

**DOI:** 10.3390/pharmaceutics15051409

**Published:** 2023-05-05

**Authors:** Christos Kaikousidis, Paraskevi Papakyriakopoulou, Aristides Dokoumetzidis, Georgia Valsami

**Affiliations:** Department of Pharmacy, National and Kapodistrian University of Athens, Panepistimiopolis, 15771 Athens, Greece; chriskaik@pharm.uoa.gr (C.K.); ppapakyr@pharm.uoa.gr (P.P.)

**Keywords:** pharmacokinetic modelling, brain pharmacokinetics, blood pharmacokinetics intranasal, NONMEM, donepezil

## Abstract

Intranasal delivery is a non-invasive mode of administration, gaining popularity due to its potential for targeted delivery to the brain. The anatomic connection of the nasal cavity with the central nervous system (CNS) is based on two nerves: olfactory and trigeminal. Moreover, the high vasculature of the respiratory area enables systemic absorption avoiding possible hepatic metabolism. Due to these physiological peculiarities of the nasal cavity, compartmental modeling for nasal formulation is considered a demanding process. For this purpose, intravenous models have been proposed, based on the fast absorption from the olfactory nerve. However, most of the sophisticated approaches are required to describe the different absorption events occurring in the nasal cavity. Donepezil was recently formulated in the form of nasal film ensuring drug delivery in both bloodstream and the brain. In this work, a three-compartment model was first developed to describe donepezil oral brain and blood pharmacokinetics. Subsequently, using parameters estimated by this model, an intranasal model was developed dividing the administered dose into three fractions, corresponding to absorption directly to the bloodstream and brain, as well as indirectly to the brain expressed through transit compartments. Hence, the models of this study aim to describe the drug flow on both occasions and quantify the direct nose-to-brain and systemic distribution.

## 1. Introduction

Central nervous system (CNS) disorders, especially neurodegenerative diseases, remain a challenging field for researchers due to their pathophysiological complexity. The development of new drug molecules is a demanding process in terms of time and resources [1]. In Alzheimer’s disease (AD), the translational gap between basic research and clinical trials considerably influences the release rate of new drugs [2], while the available oral treatments are therapeutically insufficient due to several reasons, such as the extended hepatic metabolism and limited brain targeting that can result in interindividual variability of drug responses [3,4,5,6].

Intranasal (IN) administration is a feasible alternative for brain targeting eschewing most of the limitations of oral drug delivery to the brain or blood circulation [7]. In particular, the nasal cavity is directly connected with the CNS via the olfactory (Cranial nerve I) and trigeminal (Cranial nerve V) nerves, bypassing the blood–brain barrier, while the respiratory epithelium is characterized by extended vasculature and high permeability [8]. Fast drug transfer to the blood and brain tissue after IN administration (within 5 min) enables IN delivery to be considered equivalent to the intravenous one [7]. The extracellular transport, via both nerves, follows the bulk flow and can occur rapidly [9]. The exact time needed to travel each nerve cannot be rigidly determined, as published studies mention varying durations from 5 to 45 min [10]. Furthermore, a dose fraction may also be transferred intracellularly, endocytosed by both neurons, or freely moved through the intercellular space [9]. Intracellular translocation is a slow, size-independent process that can last from one to several hours [10]. The small area of drug application, the local microbiome, and the mucociliary clearance constitute significant challenges of intranasal delivery influencing the dose accuracy and the therapeutic outcome. Hence, the type of formulation (solid or liquid), as well as its properties demonstrate a critical role in successful IN administration [7].

The pharmacokinetic (PK) model development for nasally administered drugs is a challenging process without adequate reports in the literature. The development of intravenous PK models to describe IN administration datasheets has been proposed based on the fast absorption from the olfactory nerve [11]. However, to describe the IN absorption adequately, all the transport events occurring in the different nasal cavity areas should be taken into consideration. Two approaches have been employed to explain the absorption phase: the two absorption compartments (slow and fast absorption) [12] and the transit absorption to explain the direct but slow transfer to the brain [13,14]. The direct CNS drug absorption from the respiratory region is usually considered insignificant, and it is not included in model building [14]. However, in several cases, its contribution to direct transport to the brain is equivalent to that from the olfactory area [15].

Donepezil hydrochloride is a specific reversible acetylcholinesterase inhibitor approved for the management of AD. It is a small molecule with a piperidine-based structure and high lipophilicity (Log P = 3.08–4.11) [16] and presents linear pharmacokinetics in doses ranging from 1 to 10 mg/day [17]. The high lipophilicity results in extensive distribution over the tissues, showing a volume distribution equal to 11.8 ± 1.7 L/kg and 11.6 ± 1.91 L/kg, for 5 and 10 mg doses, respectively [18]. Furthermore, the drug undergoes significant first-pass metabolism leading to a percentage of 55% of donepezil in the blood as metabolites [19]. Hence, several attempts to formulate donepezil into an intranasal delivery system have been reported in the literature and they are mostly nasal liquids (nanosuspensions, nanoemulsions) [20,21] or hydrogels [22]. The IN administration of a film for nose-to-brain delivery of donepezil was first described in a comparative PK study where nasal film administration in mice was compared with the oral delivery of donepezil solution. In this study, it was found that nasal films provided efficient delivery of donepezil to both the brain and bloodstream, even if the administered dose was 2.5 times lower than the oral one [23].

In this study, compartmental PK modeling was applied to describe donepezil PO and IN pharmacokinetic data after oral solution and nasal film administration, and to incorporate information on the physiology of the nasal cavity and the nose-to-brain connection. The developed model aims to describe drug flow after both occasions and quantify the direct and systemic nose-to-brain distribution. To the best of our knowledge, this is the first time that a semi-mechanistic PK model has been developed for an intranasal donepezil formulation.

## 2. Materials and Methods

### 2.1. Animal Experiments

The comparative PK study in eight-week-old C57BL/6J mice administered with donepezil (MW: 379.50 g/mol, Cipla Ltd., Kanpur, India) orally and intranasally is described in our recent work [23]. Briefly, the mice were divided into two groups (PO and IN) receiving 0.2 mL of the 1.25 mg/mL drug solution orally by gastrogavage (10 mg/kg), and one nasal film of 0.1 mg donepezil dose (4 mg/kg) administered intranasally. The IN administration was performed via the method described by Balafas et al. (2022) [24]. The time points of interest for measuring donepezil levels in the brain and serum were set at 5, 10, 15, 30, 45, 60, 120, 240, and 360 min (n ≥ 5 animals at every time point) after the oral treatment, and 5, 10, 15, 30, 45, 60, 90, and 120 min (n = 3–5 animals at every time point) after the IN administration. Blood samples were obtained from submandibular bleeding and then centrifuged (10,000 rpm, 15 min, 4 °C) to separate the serum, while the brain was collected after total body perfusion [25] with cold PBS pH 7.4 to remove the residual blood. Consequently, the measured concentration of brain levels corresponds to the net tissue. The donepezil amount was extracted from the serum and brain tissue samples and then quantified by using HPLC-PDA prominence system (Shimadzu, Kyoto, Japan), following the method described by Papakyriakopoulou et al. (2023) [23]. Briefly, 25 μL serum/homogenized brain sample was vortexed for 10 s, with 50 μL of the methanolic internal standard solution and 25 μL of mobile phase (50% phosphate buffer, 40% methanol, 10% acetonitrile, pH 2.8). The internal standard solution caused protein precipitation, and then it was separated by centrifugation at 10,000 rpm, 4 °C for 10 min. The supernatant was injected into the HPLC system for analysis.

### 2.2. Data Handling

The PK model was developed to fit the raw data, i.e., concentrations from all animals at all time points, as well as the mean data. In the second case, the mean value of the concentrations across all animals for each time point was calculated and the model was fit to that. This was performed in order to study the replicability of the estimated parameters between the two data representations and also assess the effect of data variability in the estimation process. The limit of quantification (LOQ) for the serum was calculated as 0.043 μg/mL, while for the brain it was 0.038 μg/mL [23]. In the modeling stages, all data below the limit of quantification (BLQ) were set to LOQ/2 [26], mainly because there were data points above but close to the LOQ for both cases, (IN and Per Os) and the model would be biased for these low values. 

### 2.3. Pharmacokinetic Model Building

Compartmental PK modeling was performed in four stages. Initially, the Per Os PK data for the blood were studied separately to obtain a first estimate for the parameters later used in the combined Per Os model, which modeled both measurements in the blood and brain simultaneously. In the second modeling stage, the blood volume of distribution was used as a fixed parameter in the total model in order to avoid identifiability issues. In this stage, classic multi-compartmental models were tested.

In the third stage, the IN dataset was modeled using parameters estimated in the Per Os modeling stages as prior knowledge. This served a double purpose as follows: first, to make the optimization procedure easier in terms of estimating fewer parameters, and second, to show the robustness of the modeling procedure since processes that are independent of the route of administration are described by the same parameter values. For the IN data regarding the brain compartment, the concentration is the total brain concentration consisting of the olfactory and the rest of the brain. In this case, more complex models were tested since the data clearly suggested a double peak phenomenon mainly attributed to a second stage of slower but direct brain absorption, which are further discussed in the following sections. Therefore, various combinations were tested, which included transit compartments in the nasal area transferring the drug to the brain region. Finally, in the fourth and last stage, a combined fitting of the model to the data was carried out describing both Per Os and IN administrations, using the models from the previous stages and the parameters as initial estimates to further validate our modeling strategy. Both additive and proportional residual error models were tested for each case. The entire analysis was carried out using the software NONMEM 7.4 (Icon Development Solutions, Hanover, MD, USA) [27]. 

Model evaluation was carried out through various numerical and graphical criteria. In each modeling stage, the model with the lowest objective function value was chosen and a visual inspection of the fit followed. For the fitting of the raw data where more data points were available, more diagnostic plots were used to validate the model. Predicted versus observed (PVO) and residuals versus time (WRES) graphs were studied in order to evaluate the goodness of fit and bias of the predictor.

## 3. Results

### 3.1. Per Os Administration

**Figure 1:**
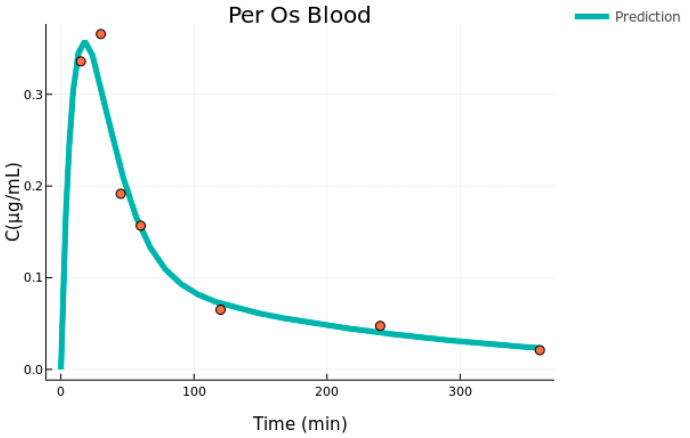
Per Os blood individual fit (mean concentration data versus time, n = 5–6).

Initially, a two-compartment model consisting of the central (blood) and a peripheral compartment was fitted to the blood data from the Per Os administration. This was done to achieve an estimate for the central volume of distribution (V_blood_), which was then used as a fixed parameter in the combined model for the Per Os dataset (blood and brain), to avoid identifiability issues. The parameter estimates alongside the standard errors and the relative standard errors for the blood fit appear in Table 1, while the graphical result is in Figure 1. The parameters are interpreted as follows: k_12_ and k_20_ are the absorption and elimination rates, respectively, while k_23_ and k_32_ are the transfer rates between the blood (1) and the peripheral (2) compartments. Finally, V_blood_ is the blood volume of distribution. The error model was proportional to b1 = 0.0117 with SE = 0.0037.

**Figure 2:**
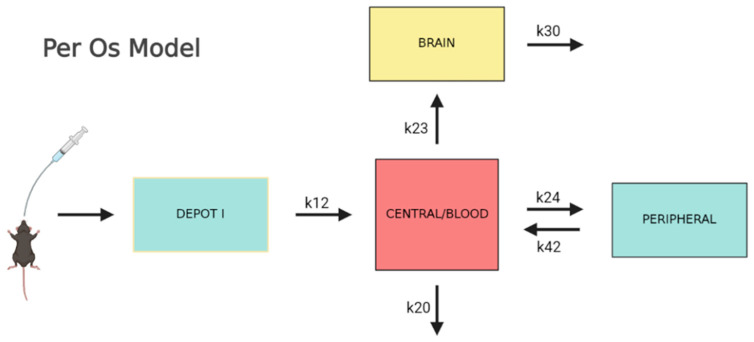
Visual description of the combined Per Os model.

In the second modeling stage, the combined blood and brain data that was found is best described by a three-compartment model which is presented in Figure 2. The drug is absorbed through a depot compartment to the blood and is transferred to and from the rest of the body (peripheral) and brain. The transfer to the brain is unidirectional since the rate k_30_ combines all the events of brain elimination, i.e., transfer to the blood, clearance to lymph nodes, as well as possible metabolism. This is identical to the parent-metabolite model, for which the metabolite volume is not identifiable [28]. In this study, the blood volume was fixed to the value given by the individual fit so the brain volume of distribution could be determined.

The fitting was performed on both the raw and mean datasets. In the second case, a mean value was calculated across all concentrations for each time point. The parameter estimates for both cases are shown in Table 2, alongside their standard errors and the relative standard errors. Besides the transfer, absorption, and elimination rates depicted in Figure 2, the drug volume of distribution in the brain is also estimated. In both cases, the parameters describing the kinetics in the central blood and its peripheral compartments are very similar to the ones estimated in the individual blood fit shown in Table 1. This proves that the fit is robust and the parameters are properly estimated. The residual error models that best describe the data are proportional for both tissues in the mean data fitting and additive for both tissues in the raw data case. Their values with their SE and RSE values appear in Table 2. 

The standard errors are relatively low, especially in the mean data estimation, which is below 22% indicating a precise estimation. For the case of the raw data, the RSE values are slightly higher which can be partly attributed to the variability of the data. Furthermore, in both models describing the mean and raw data, the estimated parameters have similar values indicating process robustness. 

**Figure 3:**
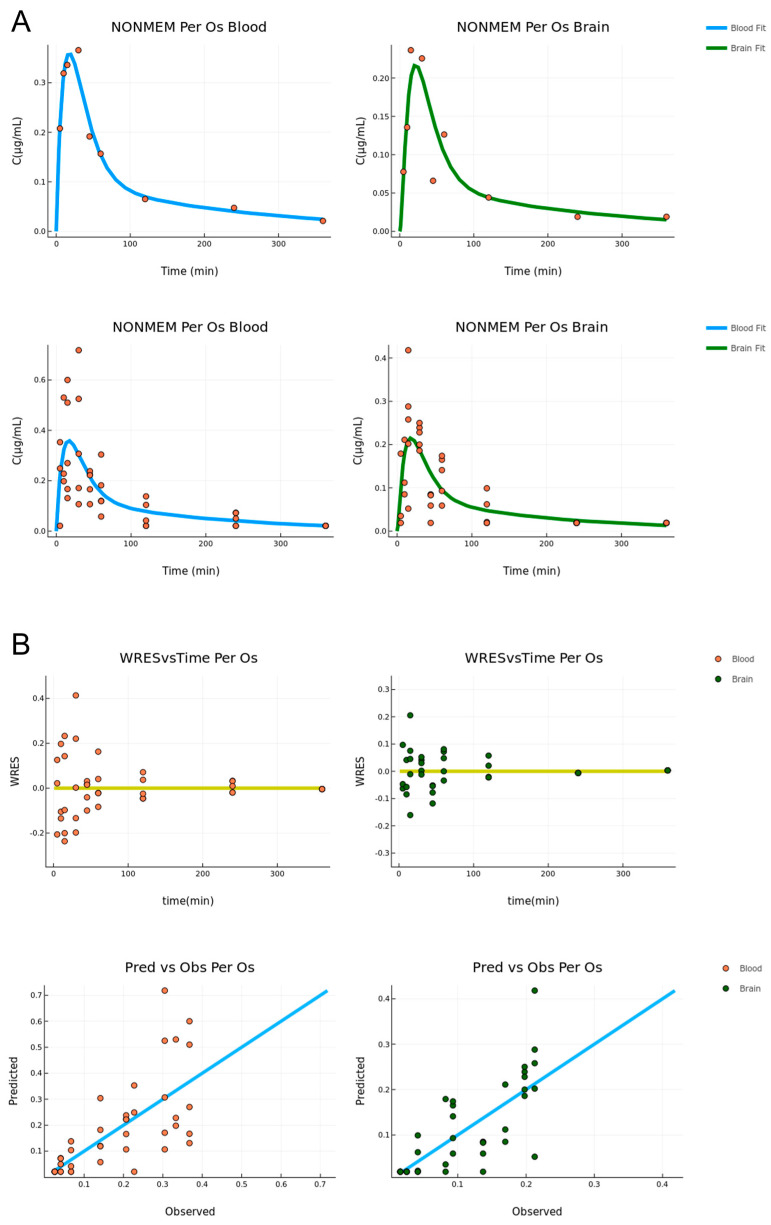
(**A**) Per Os administration combined model fit for the mean (**upper**) and raw (**bottom**) data, respectively (concentration versus time, n = 5–6). The blood and brain fit is described by the blue and green lines, respectively. (**B**) Diagnostic plots for the Per Os combined fit. Residual vs. time (**upper**) and predicted vs. observed (**bottom**) (n = 5–6). The blood and brain data are described by orange and green points, respectively.

The model fit is graphically displayed in Figure 3. First, the fit on the mean data is very adequate in the sense that it describes the trend of the data and passes through the data points for both tissues, with the exception of one point in the brain compartment (t = 45 min). The situation is the same for the fit to the raw data, for which the curve goes through the middle of the range set by the data points for each time point. The variability of the data is high for the T_max_ of both tissues; however, the model is able to describe the main data trend and still describe the mean concentration well. 

The diagnostic plots for the fitting to the raw data are displayed in Figure 3. One is the residuals versus time, and the other is the predicted versus observed. In the former, the data appear to be randomly distributed around the y = 0 axis, indicating that the source of the variability is random and the model is unbiased. In the latter, for most points, the model is reasonably accurate, and the points are randomly distributed around the linear curve. For both cases, no pattern is observed indicating an overall accurate and unbiased estimation.

### 3.2. Intranasal Administration

**Figure 4:**
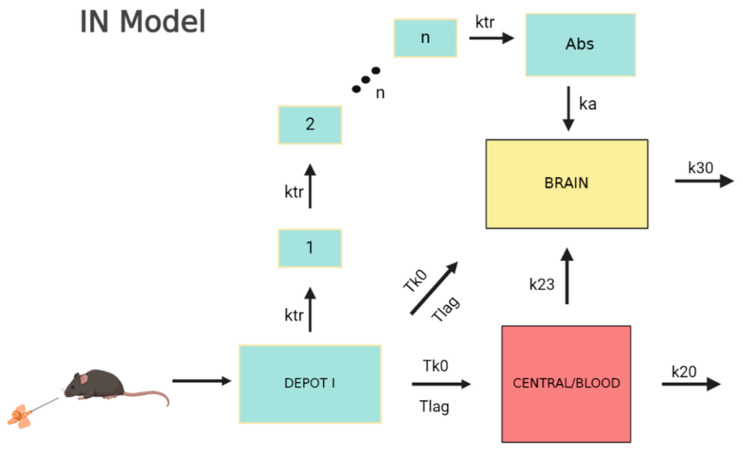
Visual description of the IN model.

In the third stage, the data from the IN administration were modeled. Following the same rationale as before, both the mean and raw data were used for the fitting. Additionally, in order to develop a more robust model and validate it in terms of coherence, information from the previous stages was used. In practice, this means that the parameters that were considered independent of the administration route were fixed (or used as priors) to the values obtained from the Per Os fitting process. The IN model was, therefore, developed with two goals in mind: to keep it in a similar form as the Per Os model where possible (i.e., blood compartment) and to describe the peculiarities arising from the IN route of administration, considering the physiology of the nasal cavity enabling the direct nose-to-brain connection. The model that best described the data is displayed in Figure 4. The dose is administered in a depot and from there it is split into three fractions: one goes directly to the circulation (blood), one directly to the brain, and one indirectly to the brain in the form of a delayed absorption expressed through transit compartments. The last fraction was implemented in the model in order to describe the second peak which was present in the brain tissue data and is discussed in the next section. The different fractions were modeled as follows: F_2_ = P_2_ the fraction for the blood compartment; F_3_ = (1 − P_2_) × P_3_ the fraction for the direct brain absorption; and F_4_ = (1 − P_2_) × (1 − P_3_), where P_2_ and P_3_ are the parameters estimated with values 0 ≤ P_2_ and P_3_ ≤ 1. Similarly to the previous stage, the transfer from brain to blood is modeled as an elimination rate. A transfer from blood to brain is also available such as in the Per Os case. In the model that best described the data, the blood-to-brain transfer (k_23_) and the brain elimination (k_20_) were fixed to the values obtained in the previous modeling stage (Per Os), while the blood elimination (k_30_) was taken as a prior but it was not fixed. The rapid absorption that was observed in both tissues was better described by a zero-order absorption with a lag time than a first-order absorption. The same lag time and absorption duration were applied to the brain and blood compartments. For the late brain absorption described by the transit compartments (estimated compartments (N) described by k_tr_ = (N − 1)/Mtt: 8.58 and 10.14, for the IN mean and raw data, respectively), the mean transit time (Mtt), the transfer rate (k_tr_) and the absorption rate in the brain (k_a_) were estimated. The estimated parameters for both cases appear in Table 3. 

**Figure 5:**
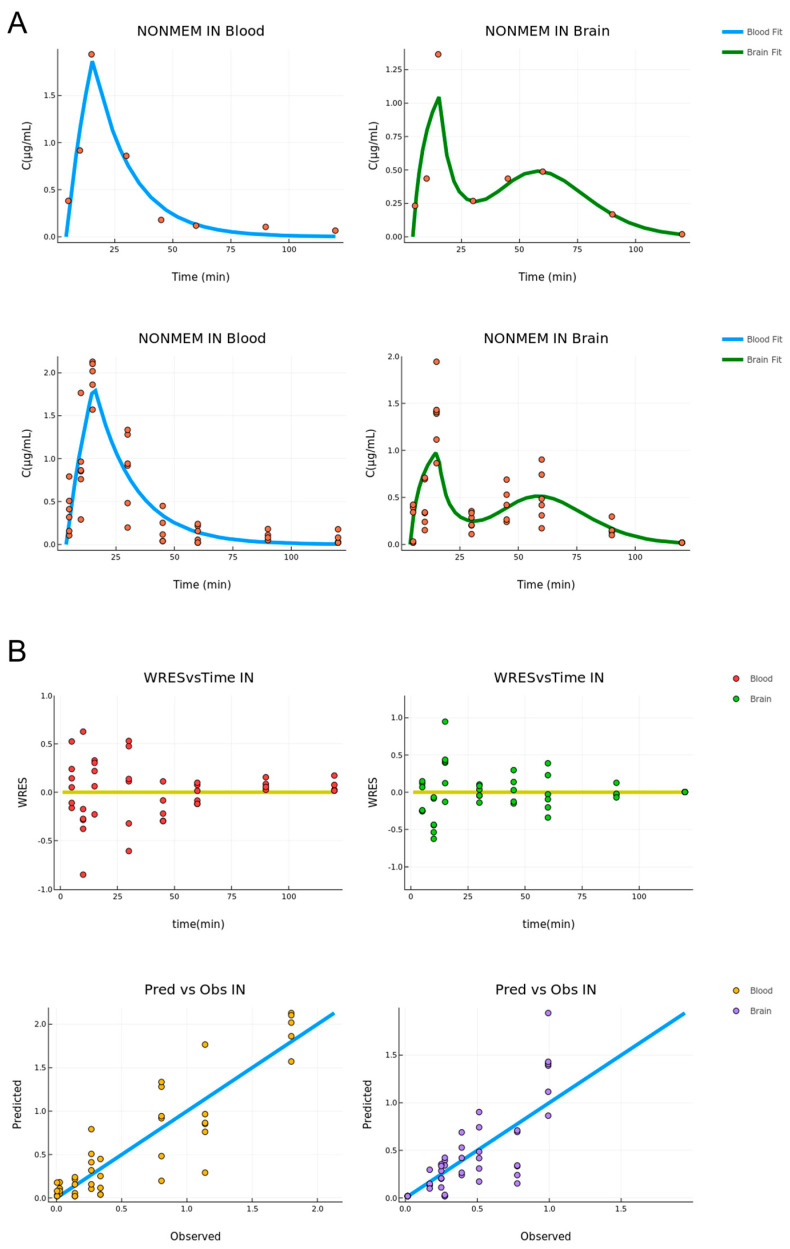
(**A**) ΙΝ administration combined model fit for the mean (**upper**) and raw (**bottom**) data, respectively (concentration versus time, n = 3–7). The blood and brain fit is described by the blue and green lines, respectively. (**B**) Diagnostic Plots for the ΙΝ combined fit. Residual vs. time (**upper**) and predicted vs. observed (**bottom**) (n = 3–7). The blood and brain data are described by orange and green points, respectively.

Once again, the parameter values in both cases were very close, with the exception of the late brain absorption rate constant (ka). Standard errors are also reasonable for an accurate parameter estimation. The residual error models for both cases (mean and raw data) were the same: an additive error model for the central compartment and a proportional error model for the brain. Their values with SE and RSE values appear in Table 3. The plots for the fit of the intra-nasal (IN) model appear in Figure 5.

For both the blood and brain tissue, the fitting describes the data well. In particular, in the case of the blood compartment, the curve passes through all the data points besides the last time points where the low concentration values are replaced by the LOQ. In the brain compartment, the model describes the main trend of the data and does especially well in the second peak appearing for t = 60 min, indicating that the transit compartment model is adequate to describe this behavior. However, this model is not able to reach the C_max_ for the first concentration peak. The diagnostic plots for the total model describing the raw data appear in Figure 5. In the predicted versus observed plots, the predicted values once again appear to be close to the observed ones and the residuals are randomly distributed around the y = 0 line. This indicates an unbiased and accurate estimation.

### 3.3. Combined Model

In the final modeling stage, a total fitting was performed using the two previous models on the data from both routes of administration. In practice, this procedure involved the two previous individual models, but instead of using fixed parameters from one to the other, the rates of blood-to-brain transfer (k_23_) and elimination (k_20_, k_30_) were set to be the same for both Per Os and IN administration and were estimated using the whole raw data (Per Os and IN) alongside the rest of the parameters. The results are presented in Table 4.

**Figure 6:**
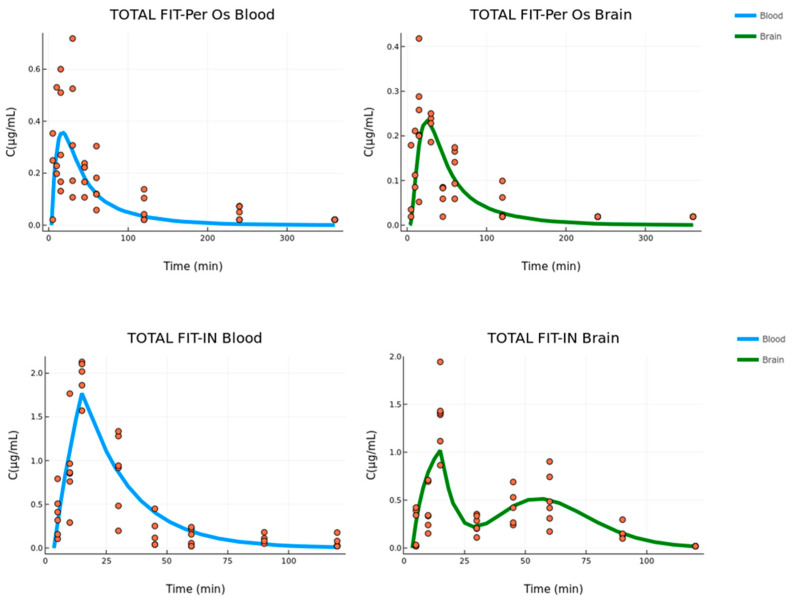
Per Os (**upper**) and IN (**bottom**) absorption fit for the combined model of the blood and brain data (concentration versus time, n = 3–7 for IN group, n = 5–6 for Per Os group). The blood and brain fit are described by the blue and green lines, respectively.

The estimated parameter values are similar to the results given in the previous modeling stages (Table 2 and Table 3) with the exception of the transit to brain absorption rate k_a_, which is significantly different (four times lower) in the total fit case. The RSE values are also higher and the parameters k_24_ and k_a_ are above 100%. However, this application is only indicative of the robustness of the modeling procedure. Hence, the estimation process was not further elaborated. The plots for the fit of the combined model appear in Figure 6.

The residual error models that best described the combined fitting were additive in the case of Per Os data for both tissues, while in the case of IN data, there was an additive error model for the central compartment and a proportional error model for the brain. The values of all residual error models with SE and RSE values appear in Table 4.

## 4. Discussion

The development of donepezil intranasal formulations has promptly enhanced in the last decade as an alternative approach for efficient and targeted delivery to CNS. The preclinical PK evaluation of these products aims to decode the events occurring in nose-to-brain and nose-to-bloodstream transfer. However, limited information on pharmacokinetic modeling has been formerly published to serve this purpose. In the present study, a four-stage compartmental PK approach was built based on the Per Os and IN data of donepezil oral solution and the recently developed nasal film [29]. The effectiveness of this nasal film compared to an oral solution on donepezil delivery in both brain and systemic circulation is depicted in C_max_ (5.7 and 3.9 times higher, respectively, at each site) and AUC values (3.1 and 1.4 times higher, respectively, at each site) obtained using the non-compartmental analysis performed in our previous work [23].

The fitting of blood and brain Per Os data revealed a three-compartment semi-mechanistic model with first-order absorption, linear elimination from the central compartment, and additional hybrid elimination from the brain considering the physiology of the nasal cavity and its direct connection to the brain. This parameter has been previously proposed by Stevens et al. (2011) [12] and is used to include all elimination procedures occurring in brain tissue, i.e., transfer to the blood, clearance to lymph nodes, as well as possible metabolism. These events, including the drug transfer from blood to the brain, are considered independent of the route of administration allowing the fixing of these parameters in IN data fitting. The blood elimination constant was let to vary in a fixed range defined by the estimated k_20_ of the Per Os model.

The high vasculature of the nasal cavity and its anatomic connection with CNS via the olfactory and trigeminal nerves can lead to the phenomenon of double absorption when the physicochemical properties (molecular weight, log P) of the administered compound permit it [30]. In several cases, the immediate systemic absorption and direct brain uptake constitute two simultaneously occurring processes, hardly delimited and distinguished into the absorption phase [12]. This issue arises especially to orally administered small molecules with adequate blood-brain-barrier (BBB) penetration that can easily reach the brain tissue when arriving in the bloodstream, but their hepatic first-pass metabolism limits the available amount for transfer to the brain [31]. The IN delivery prevents drug biotransformation by liver enzymes, while the nasal formulation can act as a drug depot that supplies concurrently to both the brain and blood compartments [12]. The donepezil blood and brain levels after nasal film administration demonstrate direct drug transfer, leading to T_max_ at the time point of 15 min, at both sites. The value of the absorption duration (T_k0_) adequately describes this phenomenon of double rapid absorption, while lag time was considered due to a slight delay (3.5–4 min) of drug release from the formulation.

In terms of kinetics, the nerve pathway consisting of olfactory and trigeminal branches can be divided into two transfer procedures with different rates ascribed to the particular position and length of each nerve. Hence, in the case of donepezil, two further absorption phases can be defined, a fast one served by the olfactory nerve and a slower one following the trigeminal nerve whose length requires a longer time to be traveled [32]. The slower drug movement is described by a series of transit compartments with an estimated Mtt of around 55–60 min. This late absorption leads to a second concentration peak observed at the respective time point of the study (60 min) and it is hypothesized that the trigeminal nerve is responsible for this phenomenon. However, the available data are not considered adequate to conclusively prove the exact source of this observation. A dose fraction of 25–30% is estimated to be absorbed by the transit compartment with k_a_ estimated equal to 0.17 and 0.57 min^−1^ for mean and raw data models, respectively. The administered dose of 20–25% is expected to undergo rapid absorption through the olfactory nerve. The contribution of blood-to-brain transfer in high tissue levels after IN administration is considered significant and refers to half of the dose, taking advantage of high donepezil permeability across the BBB and the avoidance of the first-pass effect that nasal delivery ensures [17].

Good fits were achieved in mean and raw data models for both routes of administration as it is depicted in the data distribution of PVO and WRES plots (Figure 3 and Figure 5). In the case of Per Os mean and raw data models, it was not expected to obtain identical values for the estimated parameters but of the same order of magnitude, as the error model used in each case was different. The differences reported in the estimation of a few parameters between the IN mean and raw datasheets, such as the absorption rate constant of the transit model (k_a_), are ascribed to the inability of the mean data model to take into account the interindividual variability at every time point. In particular, nasal cavity structure and components, as well as the mucosa thickness/permeability consist of physiological variants that influence absorption. Especially, in the case of the trigeminal nerve, two branches mainly participate in nose-to-brain delivery; the ophthalmic branch (V_1_) located in the olfactory region and the supraorbital maxillary branch (V_2_) in the respiratory region. Variations in length, volume, and cellular content of these branches can alter the absorption rate [9,33] and, consequently, the drug levels measured at each time point of the study.

The reliable estimation of the blood-to-brain absorption constant (k_23_), as well as of the brain elimination constant (k_30_) from the Per Os model allows the fixing of these parameters in IN model building of the third stage. In the cases of brain elimination (k_30_), blood-to-brain (k_23_), and peripheral-to-blood transfer rate (k_42_), the estimated values were not found to significantly differ among the different developed models (k_30_: 0.325 and 0.369 1/min, for Per Os mean data and raw data model, respectively; k_23_: 0.014 and 0.008 1/min, for Per Os mean data and raw data model, respectively; k_42_: 0.011 and 0.010 1/min for Per Os mean data and raw data model, respectively). This fact constitutes a strong indication of the parameters’ proper estimation, even if the %RSE of Per Os raw data model for k_30_ and k_42_ and combined data model for k_23_ are found to be high (59.35%, 61.73%, and 70.55%, respectively).

Despite donepezil being highly lipophilic, limited information on donepezil accumulation in fat tissue is reported. However, it is characterized by a high volume of distribution and long elimination half-life ranging approximately from 70 to 100 h in an age-dependent manner [4]. In particular, the PK modeling evaluation and simulation analysis of a donepezil solution in healthy male volunteers revealed a central volume of distribution equal to 456 L (~90 times higher than the blood volume of 5 L) [34]. The initial fitting for Per Os blood data, as well as the combined fitting of all Per Os data (blood and brain), resulted in V_blood_ value with a proportional difference from the physiological volume equal to 279 mL (~112 times higher than the blood volume) demonstrating the robustness of the estimation process. The volume of distribution is scaled by 1/F, where F is donepezil blood absolute bioavailability. The ten times lower value estimated after IN administration (0.021, 0.024, and 0.027 L for IN mean data model, IN raw data model, and combined data model, respectively) could be explained by the higher available dose fraction because of the first-pass effect bypassing. Correspondingly, V_brain_ is predicted to be 3 to 4 times lower (0.005, 0.003, and 0.005 L for IN mean data model, IN raw data model, and combined data model, respectively), demonstrating the positive effect of intranasal delivery in the donepezil brain exposure. 

The fourth stage of the model building combining the blood and brain data of both routes was employed as a proof of concept of parameters estimation by the individual Per Os and IN models. No significant differences are reported in the estimated parameters’ values revealed by the total fit of all datasheets. From all developed models, it is clear that the blood elimination constant, defined as k_20_, is influenced by direct nose-to-brain delivery due to the higher amount of dose reaching the systemic circulation. 

## 5. Conclusions

The compartmental modeling of blood and brain PK profiles obtained by donepezil oral solution and nasal film administration manages to adequately interpret the absorption processes governing nose-to-brain delivery. The developed IN model describing a double absorption phenomenon in the bloodstream and brain, as well as proposing two different rates (fast and slow) for nose-to-brain delivery, may be valuable for the immediate release of drugs, through nasal systems, with good BBB penetration, but extensive hepatic metabolism. The understanding of donepezil pharmacokinetics after administration in the form of the nasal film will favor the incorporation of this dosage form in the nasal delivery strategy. The developed model could be considered as a semi-mechanistic model instead of a simple compartmental model, by combining the available blood and brain data with the physiology and the processes governing nose-to-brain delivery. The future application of this model to drugs with different physicochemical properties (e.g., lipophilicity, molecular weight) will more thoroughly assess the model’s ability to describe nose-to-brain kinetics and will further highlight its applicability and possible extrapolation to humans. 

## Figures and Tables

**Table 1:** Estimated donepezil PK parameters using the Per os blood model.

Per Os Blood Data			
Parameter	Estimated Value	SE	%RSE
k_12_ (1/min)	0.064	0.004	6.43%
k_23_ (1/min)	0.032	0.003	9.78%
K_32_ (1/min)	0.012	0.003	23.64%
k_20_ (1/min)	0.024	0.001	5.61%
V_blood_ (L)	0.279	0.014	5.16%

k_12_, absorption constant; k_23_, blood-to-peripheral transfer constant; k_32_, peripheral-to-blood transfer constant; k_20_, elimination constant; V_blood_, blood volume of distribution.

**Table 2:** Estimated donepezil PK parameters using Per Os combined (blood and brain data) model.

Per Os—Mean Data			
Parameter	Estimated value	SE	%RSE
k_12_ (1/min)	0.059	0.002	3.33%
k_20_ (1/min)	0.012	0.001	6.88%
k_23_ (1/min)	0.014	0.001	5.99%
k_24_ (1/min)	0.034	0.005	13.24%
k_42_ (1/min)	0.011	0.002	20.93%
k_30_ (1/min)	0.325	0.068	21.02%
V_brain_ (L)	0.018	0.003	16.57%
**Per Os—Raw Data**			
**Parameter**	**Estimated value**	**SE**	**%RSE**
k_12_ (1/min)	0.065	0.013	20.37%
k_20_ (1/min)	0.017	0.004	22.64%
k_23_ (1/min)	0.008	0.002	19.24%
k_24_ (1/min)	0.036	0.01	28.30%
k_42_ (1/min)	0.01	0.006	61.73%
k_30_ (1/min)	0.369	0.219	59.35%
V_brain_ (L)	0.009	0.004	45.94%
**Per Os—Mean Data**			
**Parameter**	**Estimated value**	**SE**	**%RSE**
b_1_	0.012	0.004	32.02%
b_2_	0.062	0.039	62.93%
**Per Os—Raw** **Data**			
**Parameter**	**Estimated Value**	**SE**	**%RSE**
a_1_	0.016	0.005	28.48%
a_2_	0.004	0.001	31.09%

k_12_, absorption constant; k_20_, blood elimination constant; k_23_, blood-to-brain transfer constant; k_24_, blood-to- peripheral transfer constant; k_42_, peripheral-to-blood transfer constant; k_30_, brain elimination constant; V_brain_, brain volume of distribution; a_1_, additive residual error for blood; a_2_, additive residual error for brain; b_1_, proportional residual error for blood; b_2_, proportional residual error for brain.

**Table 3:** Estimated donepezil PK parameters using IN administration model.

IN—Mean Data			
Parameter	Estimated Value	SE	%RSE
T_lag_ (min)	4.06	0.465	11.45%
T_k0_ (min)	11.3	3.54	31.33%
Mtt (min)	56	1.01	1.80%
k_tr_ (1/min)	0.171	0.0176	10.29%
V_1_	0.021	0.0119	56.40%
V_2_	0.005	0.0008	16.33%
P_1_	0.543	0.131	24.13%
P_2_	0.3	0.115	38.33%
k_a_ (1/min)	0.173	0.0146	8.44%
k_20_ (1/min)	0.046	0.0229	49.78%
**IN—Raw Data**			
**Parameter**	**Estimated Value**	**SE**	**%RSE**
T_lag_ (min)	3.72	0.371	9.97%
T_k0_ (min)	11.6	4.47	38.53%
Mtt (min)	60.9	2.4	3.94%
k_tr_ (1/min)	0.183	0.025	13.50%
V_1_	0.027	0.015	55.15%
V_2_	0.003	0.001	19.28%
P_1_	0.683	0.074	10.85%
P_2_	0.276	0.108	39.13%
k_a_ (1/min)	0.566	0.128	22.61%
k_20_ (1/min)	0.05	0.021	42.69%
**Per Os—Mean Data**			
**Parameter**	**Estimated Value**	**SE**	**%RSE**
a_1_	0.016	0.006	39.81%
b_2_	0.043	0.035	80.47%
**Per Os—** **Raw Data**			
**Parameter**	**Estimated Value**	**SE**	**%RSE**
a_1_	0.082	0.022	26.09%
b_2_	0.238	0.058	24.41%

T_lag_, lag time; T_k0_, absorption duration; Mtt, mean transit time; k_tr_, transfer constant; V_blood_, blood volume of distribution; V_brain_, brain volume of distribution; P_1_, fraction of dose absorbed in blood; P_2_, fraction of dose absorbed in the brain; k_a_, brain absorption constant; k_20_, blood elimination constant; a_1_, additive residual error for blood; a_2_, additive residual error for brain; b_1_, proportional residual error for blood; b_2_, proportional residual error for brain.

**Table 4:** Estimated donepezil PK parameter values using total fitting model.

Total Fit—Per Os and IN Raw Data			
Parameter	Estimated Value	SE	% RSE
k_12_ (1/min)	0.065	0.013	19.79%
k_20_ (1/min)	0.039	0.009	23.66%
k_23_ (1/min)	0.011	0.008	70.55%
k_24_ (1/min)	0.018	0.022	119.67%
k_42_ (1/min)	0.031	0.018	59.03%
k_30_ (1/min)	0.276	0.093	33.80%
V_brain_PO_ (L)	0.015	0.008	51.43%
T_lag_ (min)	3.59	0.004	0.11%
T_k0_ (min)	11.4	0.007	0.06%
Mtt (min)	53.8	8.51	15.82%
k_tr_ (1/min)	0.205	0.088	43.12%
V_blood_IN_ (L)	0.024	0.003	12.46%
V_brain_IN_ (L)	0.005	0.001	22.85%
P_1_	0.557	0.055	9.95%
P_2_	0.306	0.057	18.59%
k_a_ (1/min)	0.127	0.131	103.15%
**Total Fit—Per Os and IN Raw data**			
**Parameter**	**Estimated Value**	**SE**	**% RSE**
a_1_	0.017	0.005	28.32%
a_2_	0.004	0.001	30.50%
a_3_	0.084	0.022	25.66%
b_1_	0.236	0.057	24.24%

k_12_, absorption constant; k_20_, blood elimination constant; k_23_, blood-to-brain transfer constant; k_24_, blood-to-peripheral transfer constant; k_42_, peripheral-to-blood transfer constant; k_30_, brain elimination constant; V_brain_PO_, brain volume of distribution from Per Os data; T_lag_, lag time; T_k0_, absorption duration; Mtt, mean transit time; k_tr_, transfer constant; V_blood_IN_, blood volume of distribution from IN data; V_brain_IN_, brain volume of distribution from IN data; P_1_, fraction of dose absorbed in blood; P_2_, fraction of dose absorbed in brain; k_a_, brain absorption constant; a_1_, additive residual error for Per OS blood data; a_2_, additive residual error for Per Os brain data; b_1_, proportional residual error for IN blood data; b_2_, proportional residual error for IN brain data.

## Data Availability

The data presented in this study are available in the present article.
